# Characterization of cognitive symptoms in post COVID-19 patients

**DOI:** 10.1007/s00406-024-01821-z

**Published:** 2024-05-13

**Authors:** Michael Ruzicka, Simone Sachenbacher, Fides Heimkes, Aline Olivia Uebleis, Susanne Karch, Fabienne Grosse-Wentrup, Gerardo Jesus Ibarra Fonseca, Nora Wunderlich, Johannes Bogner, Julia Mayerle, Michael von Bergwelt-Baildon, Peter Falkai, Marion Subklewe, Thomas Ruzicka, Christopher Benesch, Elisabeth Valdinoci, Anna Pernpruner, Anabel Thomas, Bernhard Heindl, Hans Christian Stubbe, Kristina Adorjan

**Affiliations:** 1 Department of Medicine III, Ludwig Maximilian University (LMU) University Hospital, LMU Munich, Munich, Germany; 2 Department of Psychiatry and Psychotherapy, LMU University Hospital, LMU Munich, Munich, Germany; 3 Department of Medicine IV, LMU University Hospital, LMU Munich, Munich, Germany; 4 Department of Medicine II, LMU University Hospital, LMU Munich, Munich, Germany; 5 Faculty of Medicine, Department of Psychiatry, Psychotherapy and Psychosomatics, Psychiatric University Hospital, University of Zurich, Zurich, Switzerland; 6 Stabstelle Strategische Unternehmenssteuerung, LMU Munich, Munich, Germany

**Keywords:** Post COVID-19 syndrome, Long COVID, Cognitive impairment, Depression, Brain fog, Fatigue

## Abstract

**Abstract:**

Cognitive symptoms (CS) belong to the most common manifestations of the Post COVID-19 (PC) condition. We sought to objectify CS in PC patients using routine diagnostic assessments: neurocognitive testing (NCT) and brain imaging (BI). Further, we investigated possible associations of CS with patient reported outcomes (PROs), and risk factors for developing CS. Clinical data and PROs of 315 PC patients were assessed at a mean of 6 months after SARS-CoV-2 infection. 231 (73.3%) patients reported any sort of CS. Among them, 78 underwent NCT and 55 received BI. In NCT, the cognitive domains most affected were the working memory, attention, and concentration. Nonetheless, pathological thresholds were exceeded only in few cases. Neurocognitive performance did not differ significantly between patients complaining of severe (n = 26) versus non-severe (n = 52) CS. BI findings were abnormal in 8 (14.5%) cases with CS but were most likely not related to PC. Patients reporting high severity of CS scored worse in the PHQ-9, FSS, WHOQOL-BREF, were more likely to report impaired sleep, and had a higher prevalence of psychiatric diagnoses. Overall, NCT could confirm mild impairment in some but not all PC patients with CS, while BI studies were abnormal in only few cases. CS severity did not affect NCT results, but severe CS were associated with symptoms of depression (PHQ-9), fatigue (FSS), reduced quality of life (WHOQOL-BREF) and higher prevalence of psychiatric illnesses. These findings support the importance of NCT, BI, and neuro-psychological assessment in the work-up of PC patients reporting CS.

**Trial registration:**

Trial registration number and date of registration: DRKS00030974, 22 Dec 2022, retrospectively registered.

**Supplementary Information:**

The online version contains supplementary material available at 10.1007/s00406-024-01821-z.

## Introduction

Coronavirus disease 2019 (COVID-19) is a novel condition caused by the severe acute respiratory syndrome coronavirus 2 (SARS-CoV-2). Due to its rapid global spread after first being described in Wuhan, China in 2019, COVID-19 was declared a pandemic by the World Health Organization (WHO) in March, 2020 [1]. The Post COVID-19 (PC) condition is characterized by ongoing or the emergence of newly developing and otherwise inexplicable symptoms up to 3 months after the suspected or confirmed initial SARS-CoV-2 infection [2] and has gained growing attention over the past three years. A broad range of post-acute disease manifestations has been described, involving mainly the pulmonary, cardiovascular, hematologic, neuropsychiatric, renal, endocrine, gastrointestinal/hepatobiliary and integumentary organ systems [3]. A large meta-analysis by O’Mahoney et al. revealed that at least 45% of COVID-19 patients reported one or more unresolved symptoms after a mean follow-up time of 126 days, regardless of hospitalization status during the acute infection [4]. Another meta-analysis by the European Centre for Disease Prevention and Control (ECDC) estimates the prevalence of any PC condition symptom > 12 weeks after SARS-CoV-2 infection at 50.6% among patients recruited in community settings, 66.5% among patients recruited in hospital settings and 73.8% among ICU patients [5]. In contrast, the WHO concludes that 10–20% of COVID-19 patients develop a PC condition, estimating a number as high as 17 million people within the WHO European Region to have experienced a PC syndrome during the years of 2020–2021 [2].

Among PC patients, cognitive symptoms belong to the most frequently reported complaints (22–51.1% of PC-patients, [6–8]) and so far have been described to mainly affect concentration/attention, memory [9], receptive language and/or executive function [3]. Further, patients seem to be at higher risk of developing psychiatric diseases (i.e. anxiety disorder, mood disorder/depression, insomnia) after infection with SARS-CoV-2 compared to influenza or other respiratory pathogens [3] or control cohorts surviving sepsis caused by different pathogens than SARS-CoV-2 [10]. Despite a high prevalence of neuropsychiatric manifestations in PC patients, the underlying pathomechanisms remain subject of ongoing scientific research and discussion and include direct viral infection of the central nervous system (CNS), severe systemic or neuroinflammation, microvascular thrombosis, neurodegeneration, but also deconditioning, post-traumatic stress disorder (PTSD) and others [3]. Studies investigating possible neuroradiological correlates for neuropsychological alterations in PC patients describe subtle white matter abnormalities of the brain [11], decreased grey matter volume [12, 13], reduction of global brain size, and tissue damage in brain regions functionally connected to the primary olfactory cortex [14].

In our study, we sought to further characterize the nature of cognitive symptoms (CS) reported by PC patients by assessing various cognitive performance areas and identify possible risk factors for developing long-term cognitive disability. Further, we investigated whether neuroimaging via magnetic resonance imaging (MRI) or computerized tomography (CT) of the brain would reveal structural correlates for the reported disabilities. We sought to test the following three hypotheses:

1. CS can be objectified by routine medical assessments (neurocognitive testing (NCT), brain imaging).
2. Symptom severity of CS can be objectified by NCT.
3. Higher severity of CS is associated with worse patient reported outcomes (PROs, e.g. WHO Quality of Life Assessment).

## Patients and methods

### Post-COVID^LMU^ and patient inclusion

The Post-COVID^LMU^ outpatient clinic is an interdisciplinary department specialized on the treatment of PC patients. At first patients are examined by a specialist for internal medicine and a psychologist or psychiatrist. Depending on the individual needs, patients are then referred to colleagues of other departments involved in the Post-COVID^LMU^ network (infectiology, pneumology, cardiology, endocrinology, neurology, physical and rehabilitative medicine, others). Upon written informed consent, patients presenting to our Post-COVID^LMU^ outpatient department were included into our study if a Post COVID-19 condition according to the WHO definition [2] could be confirmed or was highly suspected. The past SARS-CoV-2 infection had to be confirmed by PCR-testing. Patients were only included into the study if the diagnosis of the acute SARS-CoV-2 infection was made within the last 4–12 months.

### Determination of SARS-CoV-2 virus strains

To assess the SARS-CoV-2 virus strains for the acute infection, time points of the respective PCRs were plotted (Supplementary Fig. 1). Based on these and after comparison to data by the Robert Koch Institut (RKI) on the prevalence of SARS-CoV-2 variants in Germany over time [15], the virus strains most likely for the acute infection were determined.

### Definition of cognitive symptoms and assessment of symptom severity

In this work, the term “cognitive symptoms” (CS) comprises the following patient-reported symptoms: impaired alertness/concentration and/or confusion and/or memory impairment and/or speech disorders. The symptoms had to occur at ≥ 3 days per week and cause an impairment of everyday life.

All patients included into the study answered standardized digital questionnaires. Among other items, the prevalence of various symptoms (including cognitive symptoms) related to the PC condition were assessed. Intensity/severity of cognitive symptoms was rated by patients on a four-point Likert scale as none, mild, moderate, or high. In the following, the intensity of CS will be reflected by the abbreviations miCS (mild), moCS (moderate) and hiCS (high). The minimum duration of CS was determined as ≥ 2 months.

### Selection of patients for diagnostic procedures

All patients complaining of CS were offered neurocognitive testing (NCT) and brain imaging independently of symptom severity. The decision whether to perform CT versus MRI was based upon the availability of the procedure within a clinically appropriate time frame. The assessments were no prerequisite for study inclusion and performed if the patients agreed. NCT and brain imaging data were both raised within a time frame of 8 weeks.

### Neurocognitive testing

NCT refers to a set of neuropsychological clinical assessments and includes the following:

- Vocabulary tests (VT; [16])
- The Repeatable Battery for the Assessment of Neuropsychological Status (RBANS; [17])
- Trail Making Test (TMT; [18])—part A (TMT-A) and part B (TMT-B)
- Letter-Number-Span (LNS; [19])
- d2-R Test of Attention (d2-R; [20]).

VT was performed to assess the patients’ verbal intelligence and estimate the premorbid intelligence quotient (IQ) as laid out by Lehrl et al. [21]. RBANS is a tool to measure the following five cognitive performance areas using the subsequently listed subtests:

- Immediate memory: List Learning (LL), Memorized Words in the 4th Round (MW4), Story Memory (SM)
- Delayed memory: List Recall (LR), List Recognition (LRg), Story Recall (SR), Figure Recall (FR)
- Visuospatial function/construction: Figure Copy (FC), Line Orientation (LO)
- Attention: Digit Span (DS), Coding/Symbol-Number-Test (SNT)
- Language: Semantic Fluency (SF), Picture Naming (PN)

TMT assesses visuomotoric processing speed, cognitive flexibility and working memory, while LNS provides information about the working memory. Lastly, d2-R assesses the domains of attention and concentration. Supplementary Table 1 provides an overview and further details on the assessments. If not indicated differently, results are depicted as age normalized standardized values (ASVs) with a standard value of 100. ASVs of < 85 are considered pathological.

### Patient-reported outcome measures (PROMs)

Patients digitally answered clinical questionnaires including the WHO Quality of Life Assessment (WHOQoL-BREF; [22]) and were screened for depressive symptoms by means of the 9-item Patient Health Questionnaire (PHQ-9; [23, 24]). Levels of fatigue were assessed using the Fatigue Severity Scale (FSS; [25, 26]). PROMs were raised at the time point of study inclusion.

###  Definition and evaluation of insomnia

Each patient was screened for signs of insomnia by their attending physician. For this study, the symptom insomnia was defined in accordance with the ICD-10 definition of nonorganic insomnia (F51.0) [27]. It was further enhanced based on the definition by Roth, T. [28] as a condition with:

1. Difficulty falling asleep, staying asleep or nonrestorative sleep.
2. These difficulties occurring despite adequate opportunity and circumstance to sleep.
3. These sleeping disorders being associated with daytime impairment or distress.
4. A minimum frequency of sleep difficulties of 3 times per week and an overall duration of ≥ 2 months.

### Brain imaging

Brain imaging (MRI, CT) was offered to all patients complaining of CS and performed if patients agreed. CT was performed using a dual-source CT scanner. MRIs were conducted by means of 1.5- or 3.0-T clinical MRI scanners. MRI protocols included the following sequences: axial diffusion weighted imaging (DWI; slice thickness (ST): 5mm), axial 3D fluid attenuated inversion recovery (FLAIR) with reconstruction in coronal and sagittal planes (ST: 3mm), axial T2-weighted heme (ST: 3mm), axial T2-weighted turbo spin echo (TSE; ST 3mm), T1-weighted axial imaging (ST: 3mm), and time-of-flight magnetic resonance angiography (TOF MRA). CT and MRI scans were analyzed by the attending and validated by a senior radiologist. Both were blinded regarding the intensity of CS. The image analysis and the respective reports followed a standardized clinical assessment protocol. All abnormal deviations with or without known pathological meaning were reported.

### Data acquisition

Clinical, NCT and radiological/imaging data as well as patient reported symptoms were collected using the lightweight clinical data acquisition and management software for clinical research (LCARS-C, LMU Munich).

### Statistical analyses

Statistical analyses were performed using R Studio version 4.2.1.

Medians with interquartile ranges (IQR) are shown for numeric variables, whereas categorical variables are listed as absolute counts with their respective percentages.

Statistical significance between the medians of groups was calculated using a two-sided Kruskal–Wallis test. Statistical differences between count data were assessed by means of the Pearson’s Chi-squared test.

ASVs of NCT were compared to their respective standard values [age normalized standardized standard values (ASSV)] using a one-sided one-sample t-test, testing the hypothesis of a diminished neurocognitive performance.

Results are displayed along their 95% confidence intervals (CI). Statistical differences were considered significant at p-values < 0.05. p-values of NCT were adjusted for multiple comparisons using the Benjamini–Hochberg method.

## Results

**Fig. 1:**
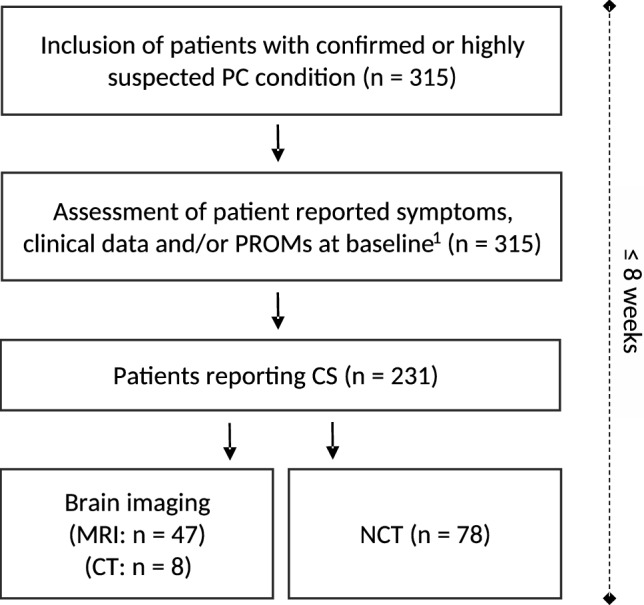
Flow chart of the study design. ^1^Baseline resembles the time point of study inclusion

We assessed baseline data comprised of patient reported symptoms, clinical data reported by attending physicians, and PROMs in a total number of 315 patients with a confirmed or highly suspected PC condition in our study (Fig. 1). The median time to presentation to our outpatient department was 183 [124;318] days after the initial SARS-CoV-2 infection. 80.3% of the patients complained of impaired alertness, 68.6% of confusion, 64.4% of memory impairment and 35.1% of speech disorders (Table 1); these symptoms are referred to as CS in the following. Further, 87.2% of patients reported fatigue, 57.4% insomnia, 53.2% anxiety or strain and 47.9% a depressive mood. The impairment of daily life as a consequence of each reported symptom was described as intermediate or even severe in more than 50% of the cases (Table 1). The frequency of concentration difficulties was described as daily by 26.5% of the patients while 24.7% reported the issues to occur on more than half of the days. Only 14.1% of patients negated concentration issues. Of 231 patients complaining of any sort of CS, 55 were further evaluated by means of brain imaging and 78 underwent NCT (Fig. 1).

**Table 1:** Neuropsychiatric symptoms reported by Post COVID-19 patients

	Patients affected^a^ (total: n = 315)	Impairment of everyday life^a^		
		Mild	Intermediate	Severe
Impaired alertness	151 (80.3%)	26 (17.6%)	68 (45.9%)	54 (36.5%)
Confusion	129 (68.6%)	13 (23.2%)	33 (58.9%)	10 (17.9%)
Memory impairment	121 (64.4%)	39 (32.2%)	60 (49.6%)	22 (18.2%)
Impaired speech	66 (35.1%)	31 (47.7%)	28 (43.1%)	6 (9.23%)
Fatigue	164 (87.2%)	15 (9.26%)	49 (30.2%)	98 (60.5%)
Insomnia	108 (57.4%)	16 (15.0%)	53 (49.5%)	38 (35.5%)
Anxiety or strain	100 (53.2%)	23 (23.2%)	42 (42.4%)	34 (34.3%)
Depressive mood	90 (47.9%)	30 (34.1%)	39 (44.3%)	19 (21.6%)

^a^Absolute numbers are followed by the respective percentages. Percentages refer to percent of patients who answered the respective question in the digital patient health report form of the LCARS-C, not to percent of total patients included in the study

**Fig. 2:**
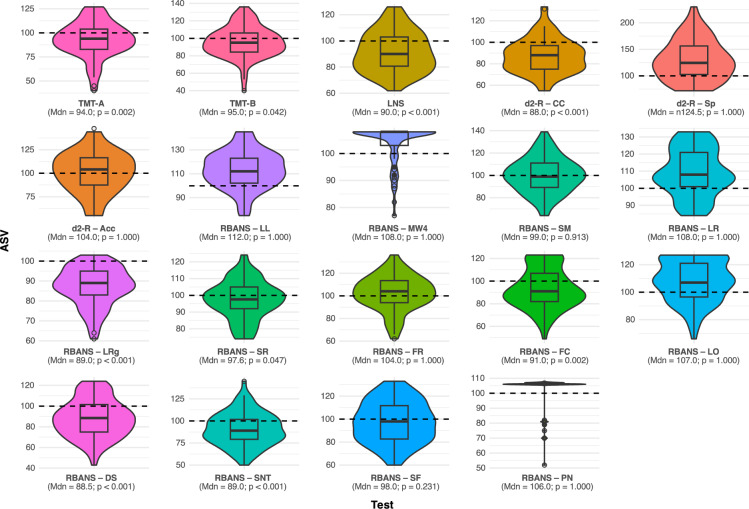
Results of neurocognitive testing in PC patients reporting cognitive symptoms. ASV = age normalized standardized value (standard value = 100); Mdn = median. Standard values are reflected by the dashed, median values by bold horizontal lines; IQR is indicated by the respective rectangles; Dots resemble outliers beyond the 90th or 10th percentile, respectively

NCT was performed to further classify the areas of neurocognitive dysfunction (Fig. 2). The outcomes of the TMT part A and B show slight impairment across both tests (median ASV for TMT-A: 94.0, p = 0.002; median ASV for TMT-B: 95.0, p = 0.042), while the results of the LNS (median ASV: 90.0, p < 0.001) and the d2-R section examining concentration capacity (d2-R—CC; median ASV: 88.0, p < 0.001) display more pronounced negative deviations from the reference values. Importantly though, the pathologic threshold (ASV of 85) was not underrun in any of the assessments by the median values. Further, neither speed (d2-R—Sp) nor accuracy (d2-R—Acc) of d2-R testing were negatively impaired (p = 1.000 for both subtests). Lastly, the RBANS showed mixed results. The sections List Learning (LL), Memorized Words in the Fourth Round (MW4), Story Memory (SM), List Recall (LR), Figure Recall (FR), Line Orientation (LO), Semantic Fluency (SF), and Picture Naming (PN) were statistically not significantly impaired (p > 0.05, compare Fig. 2). In contrast, we found the sections List Recognition (LRg), Story Recall (SR), Figure Copy (FC), Digit Span (DS) and Coding/Symbol-Number-Test (SNT) to be negatively affected to a statistically significant extent. Again, none of the median ASVs of RBANS testing fell below the pathologic benchmark.

Supplementary Table 2 provides an overview over the number of patients whose NCT results dropped below the first negative standard deviation (−1 SD), which reflects the pathologic threshold of the respective subtests. The assessments with the highest number of patients below −1 SD were d2-R-CC [n = 34 (45.3%)], RBANS-DS [n = 37 (47.4%)] and RBANS-SNT [n = 34 (43.6%)]. The trends are in line with the group results displayed in Fig. 2.

Next, we assessed differences between patients complaining of high (n = 56) and patients reporting no, mild or moderate levels of CS (n = 259; Table 2). The median time to presentation in our outpatient department was 171 or 184 days, respectively (p = 0.626), and the median age at inclusion was 40.5 vs 41.0 years (p = 0.988). Female patients appeared overrepresented in both groups (66.1% or 61.0%, respectively). The ethnicity of 99.7% of all patients included in the study was European with no significant differences between the two groups.

**Table 2:** Characteristics of PC patients reporting high vs lower levels of cognitive symptoms

Subjective level of cognitive symptoms^a^	High (n = 56)	None, mild, moderate (n = 259)	p Value
Patient characteristics			
Time point of first assessment (days)^b^	171 [118;315]	184 [125;320]	0.626
Age at inclusion (years)	40.5 [34.0;54.0]	41.0 [31.5;53.0]	0.988
Sex at birth			0.578
Female	37 (66.1%)	158 (61.0%)	
Male	19 (33.9%)	101 (39.0%)	
Hospitalization status during acute SARS-CoV-2 infection			0.821
Inpatient	5 (9.3%)	18 (7.6%)	
Outpatient	49 (90.7%)	217 (91.9%)	
Unknown	0 (0.00%)	1 (0.42%)	
Severity of acute infection (LEOSS classification)			0.144
Complicated	5 (9.1%)	6 (2.6%)	
Critical	0 (0.0%)	3 (1.3%)	
Uncomplicated	50 (90.9%)	218 (95.2%)	
Unknown	0 (0.0%)	2 (0.9%)	
Vaccination status^c^			0.472
BI plus one booster	15 (28.8%)	80 (34.5%)	
BI plus two boosters	1 (1.9%)	3 (1.3%)	
Complete BI	12 (23.1%)	54 (23.3%)	
Incomplete BI	4 (7.7%)	8 (3.5%)	
Not vaccinated	12 (23.1%)	64 (27.6%)	
Unknown	8 (15.4%)	23 (9.9%)	
No. of previous SARS-CoV-2 infections	1.00 [1.00;1.00]	1.00 [1.00;1.00]	0.960
Insomnia	23 (41.1%)	51 (20.3%)	0.002
PROMs			
WHO physical health (points)	32.1 [17.9;43.8]	50.0 [35.7;64.3]	< 0.001
WHO psychological health (points)	45.8 [33.3;54.2]	62.5 [45.8;70.8]	< 0.001
WHO social relationship (points)	66.7 [50.0;75.0]	70.8 [58.3;75.0]	0.070
WHO environment (points)	65.6 [56.2;75.0]	75.0 [65.6;81.2]	< 0.001
PHQ-9 Score (points)	15.0 [11.2;17.0]	8.0 [6.0;12.0]	< 0.001
Fatigue Severity Scale (score)	58.5 [56.6;62.0]	51.0 [41.8;59.0]	< 0.001
Psychiatric diagnoses (PD)			
Patients with PD total	26 (46.4%)	71 (27.4%)	0.008
Patients with pre-existing^d^ PD	23 (41.1%)	64 (24.7%)	0.020
Patients with newly diagnosed^e^ PD	7 (12.5%)	12 (4.6%)	0.055

*BI* Basic immunization, implying the administration of two doses of SARS-CoV-2 vaccine in a time interval specified by the respective manufacturer

^a^Based on a four-point Likert scale as specified in “Patients and Methods”

^b^Refers to the time point of the first presentation to our outpatient department in days after the primary SARS-CoV-2 infection

^c^Vaccination status at the time point of the initial SARS-CoV-2 infection

^d^Pre-existing PD refers to psychiatric conditions diagnosed before the initial SARS-CoV-2 infection

^e^Newly diagnosed PD comprise conditions diagnosed after the initial SARS-CoV-2 infection and before study inclusion

The reported intensity of CS did not differ based on the hospitalization status (inpatient vs outpatient) during the acute SARS-CoV-2 infection (p = 0.821; Table 2). The vast majority of acute infections (> 90% for both groups) had taken an “uncomplicated” course according to the Definition of Clinical Phases by the Lean European Open Survey on SARS-CoV-2 infected patients (LEOSS, [29]). Based on this classification, no statistically significant differences between the two groups were detected with regard to disease severity during the acute course of disease.

In both groups, > 50% of patients had at least completed their basic SARS-CoV-2 immunization or had received additional booster vaccinations at the time point of the initial SARS-CoV-2 infection (Table 2). About one fourth of patients (23.1% or 27.6%, respectively) were not vaccinated at all. Between the two groups, we did not detect any statistically significant differences with regard to vaccination status (p = 0.472) or number of previous SARS-CoV-2 infections (p = 0.960). Based on the distribution of SARS-CoV-2 PCRs confirming the acute infection over time (Supplementary Fig. 1), early subtypes of the Omicron (B.1.1.529) virus strain followed by Delta (B.1.617.2) appear to be the most prevalent ones amongst our patients. The distribution over time is even between the groups of patients reporting hiCS versus lower intensity of CS. Consequently, we do not expect significant differences in the virus strains responsible for the acute infection between those groups.

Interestingly, sleeping disorders/insomnia appeared to be more prevalent in patients complaining of hiCS compared to lower intensity of CS (41.1% vs 20.3%, p = 0.002; Table 2). Patients with hiCS scored significantly worse in multiple components of the WHOQoL-BREF, namely the physical health (32.1 vs 50.0 points, p < 0.001), psychological health (45.8 vs 62.5 points, p < 0.001) and environment (65.6 vs 75.0 points, p < 0.001) section. Further, those patients reached higher scores when screened for symptoms of depression by means of the PHQ-9 (15.0 vs 8.00 points, indicating moderately severe vs mild depressive symptoms, p < 0.001) and scored higher in the FSS (58.5 vs. 51.0, p < 0.001).

Considering the patients’ previous medical record, we found that at the time of study inclusion, psychiatric diagnoses (PD) were more prevalent among patients with hiCS (46.4% vs 27.4%, p = 0.008; Table 2). Most of those patients had acquired the PD before the initial SARS-CoV-2 infection, with patients reporting hiCS again being overrepresented (41.1.% vs 24.7%, p = 0.020). The most frequent PD acquired before infection were clinical depression, followed by anxiety disorders, adjustment disorders and posttraumatic stress disorder (PTSD). PD with an onset after SARS-CoV-2 infection were also more common among patients complaining of hiCS (12.5% vs 4.6%), even though the comparison failed to reach the mark of statistical significance (p = 0.055). The most common PD acquired after SARS-CoV-2 infection and before study inclusion were clinical depression, followed by adjustment disorders, anxiety disorders and PTSD.

We next compared NCT results of patients who reported high (n = 26) to those who reported mild or moderate levels of CS (n = 52; Table 3). There was no significant age difference between those two groups (43.5 vs 48.5 years, p = 0.895). Again, women appeared overrepresented in both groups (61.5% and 55.8%, respectively). The median values of the estimated premorbid IQ based on verbal intelligence were in line with the general population (IQ = 101 or 104, respectively) and did not differ significantly between the two groups (p = 0.418). Within the RBANS we found no statistically significant differences between the two groups in any of the subtests (Supplementary Table 3). Similarly, results of the TMT part A (ASV = 89.0 vs 96.5, p = 0.191), TMT part B (ASV = 90.0 vs 95.0, p = 0.563) as well as the LNS (ASV = 85.0 vs 94.0, p = 0.191) did not differ to a statistically significant extent (Table 3). Lastly, none of the three d2-R sections revealed any statistically significant differences between the two groups, with concentration capacity being evenly reduced (ASV = 84.0 vs 88.0, p = 0.667) while the speed of operation was evenly increased (ASV 123.0 vs 124.0, p = 0.929) and accuracy (ASV = 106.0 vs 102.0, p = 0.929) close to reference values. Of note, within this subgroup analysis, the median ASV of patients with hiCS did in fact breach the pathologic cutoff for both RBANS-DS (median ASV: 80.5) and RBANS-SNT (median ASV: 84.0).

**Table 3:** Characteristics and results of PC patients undergoing neurocognitive testing

Subjective level of cognitive symptoms^a^	High (n = 26)	Mild or moderate (n = 52)	p Value
Patient characteristics			
Age at inclusion (years)	43.5 [37.8;54.8]	48.5 [35.0;55.5]	0.895
Sex at birth			
Female	16 (61.5%)	29 (55.8%)	
Male	10 (38.5%)	23 (44.2%)	0.808
Neurocognitive testing			
Estimated premorbid IQ^b^	101 [93;110]	104 [97;114]	0.598
RBANS (all subtests)^c^			*n.s*
TMT part A^d^ (ASV)	89.0 [75.2;96.8]	96.5 [86.5;108.0]	0.191
TMT part B^e^ (ASV)	90.0 [83.0;106.0]	95.0 [86.0;106.0]	0.563
LNS^f^ (ASV)	85.0 [72.0;99.0]	94.0 [81.0;108.0]	0.191
d2-R concentration^g^ (ASV)	84.0 [71.5;102.0]	88.0 [78.0;97.0]	0.667
d2-R speed^h^ (ASV)	123.0 [92.8;163.0]	124.0 [107.0;148.0]	0.929
d2-R accuracy^i^ (ASV)	106.0 [83.8;118.0]	102.0 [89.0;116.0]	0.929
PROMs			
WHOQoL-BREF: physical health section (points)	32.1 [21.4;46.4]	51.8 [33.0;68.8]	0.011
WHOQoL-BREF: psychological health section (points)	50.0 [41.7;54.2]	50.0 [44.8;58.3]	0.429
WHOQoL-BREF: social relationship section (points)	66.7 [58.3;75.0]	66.7 [47.9;75.0]	0.957
WHOQoL-BREF: environment section (points)	62.5 [56.2;75.0]	75.0 [68.5;81.2]	0.114
PHQ-9 score (points)	15.0 [10.8;17.2]	10.0 [8.0;15.0]	0.020
Fatigue Severity Scale (score)	58.0 [56.2;61.0]	52.5 [43.8;58.2]	0.038

*ASV* age normalized standardized value (standard value = 100), *n.s.* not significant

^a^Based on a four-point Likert scale as specified in “Patients and Methods”

^b^Based on verbal intelligence/vocabulary tests

^c^For detailed RBANS results, see Supplementary Table 3; Cognitive domains assessed:

^d^Visuomotoric speed

^e^Visuomotoric speed, cognitive flexibility and working memory

^f^Working memory

^g^Concentration capacity

^h^Speed of operation

^i^Accuracy

Interestingly, the most prominent differences between the two groups can be seen within the WHOQoL-BREF, PHQ-9 and FSS (Table 3). Patients experiencing hiCS performed worse in the physical health section of the WHOQoL-BREF (32.1 vs 51.8 points, p = 0.011). Further, this group reached higher scores when screened for symptoms of depression in the PHQ-9 (15.0 points vs 10.0 points, indicating moderately severe vs moderate depressive symptoms, p = 0.020). Lastly, levels of fatigue also appeared higher in this group as reflected by the FSS scores of 58.0 vs 52.5 points (p = 0.038).

Beyond cognitive performance, we assessed if there were any structural correlates for the reported CS detectable by neuroimaging (Table 4). 55 Patients who complained of CS underwent radiological imaging of the brain (n = 49 for MRI, n = 6 for CT) either due to CS themselves or in combination with different reported symptoms (i.e. headaches, nausea). 8 (14.5%) imaging results showed pathological findings [meningioma, multiple sclerosis (MS), aortic aneurysm, nasal polyps, white matter lesions (WML), lesions without pathological meaning (each n = 1), unknown (n = 2)]. Apart from both the MS and WML finding, none of the known results seem to provide a causal explanation for CS or at least they were not pronounced enough to do so. The remaining 47 (85.5%) radiological assessments showed normal findings.

**Table 4:** Radiological findings

Assessment	Pathological findings in n (%) of assessments	Normal findings in n (%) of assessments	Diagnosis	
Brain MRI (n = 49) or brain CT (n = 6)	8 (14.5)	47 (85.5)	Meningioma	(n = 1)
			Multiple sclerosis	(n = 1)
			Aortic aneurysm	(n = 1)
			Nasal polyps	(n = 1)
			White matter lesions	(n = 1)
			Lesions w/o pathological meaning	(n = 1)
			Unknown	(n = 2)

## Discussion

Neurocognitive manifestations are among the most frequent symptoms of the PC condition [3, 6–8]. Nevertheless, the exact cognitive domains affected, the level of cognitive disability as well as the underlying pathomechanisms remain elusive. In our study, we sought to provide qualitative insight into the neurocognitive affection of PC patients by identifying the cognitive domains affected, quantify the level of impairment, assess possible neurostructural correlates, and identify risk factors for the development of long-lasting cognitive dysfunction.

Overall, NCT of our patients revealed slight impairment of certain cognitive performance areas that reached levels of statistical significance. The areas most affected seem to be the working memory (LNS), attention (RBANS-DS, RBANS-SNT) as well as concentration (d2-R—CC). However, the pathological threshold (ASV < 85) of the respective tests was not underscored but in one exception: patients complaining of hiCS did indeed show pathologic impairment of attention (RBANS—DS, RBANS—SNT). In contrast, the cognitive domains of immediate memory and language did not seem to be negatively affected at all, visuomotoric speed appeared to be only marginally impaired, and the results of tests assessing delayed memory and visuospatial function showed mixed results. Interestingly, patients seemed to overperform in some cognitive areas of the d2-R and RBANS, i.e. with regard to immediate memory. However, we did not test for statistical significance of overperformance in NCT since the relevance and clinical implications remain questionable.

Taken together, most of the NCT results were within a non-pathological range and the overall mild negative deviations from the respective reference values did not underrun pathologic cutoffs in most cases. Further, NCT could not objectify any statistically significant differences between patients who reported hiCS versus miCS or moCS, suggesting that the perception of neurocognitive manifestations may be subject to confounding from several factors. Our findings are in line with the work of Dressing et al. who describes mild to no significant impairment in PC patients (n = 31) depending on the cognitive area using a neuropsychologic test battery [30]. In contrast, Ariza et al. found that PC patients (n = 319) with and without subjective cognitive complaints scored significantly worse on tests assessing multiple cognitive performance areas, namely global cognition, learning, long-term memory, processing speed, language and executive functions than healthy controls (n = 109) [31]. Further authors describe attention/processing speed [32] or attention, executive function, and memory impairment [33] across PC patients as the most frequently impaired cognitive domains based on various forms of NCT. With this in mind, the composition of our NCT protocol must be viewed as a possible limitation of our study. Based on our findings and the published literature, the addition of tests more sensitive to attention deficits and processing speed may enhance the detection of PC associated deficits.

Another limitation of our NCT protocol is the lack of a control group, which could consist of PC patients complaining of no CS at all or controls not suffering from a PC condition. The comparison of NCT findings to ASSV may be helpful to estimate a patient’s neurocognitive performance status, but even findings within or above the age adjusted norm cannot rule out a decrease of neurocognitive functions within a single individual with premorbidly above-average neurocognitive capacity. Likewise, negative deviations from the ASSV do not necessarily indicate neurocognitive decline in patients with premorbidly under-average neurocognitive capabilities. Estimates of the premorbid IQ based on VT as performed in this study may provide a basic idea of neurocognitive capacities before SARS-CoV-2 infection. However, only the direct comparison of detailed NCT results before and after SARS-CoV-2 infection would allow most precise conclusions with respect to possible cognitive decline over time / after SARS-CoV-2 infection. Thus, NCT as performed in our study appears to be of limited use as a primary diagnostic tool when it comes to the quantification of neurocognitive complaints since it usually does not allow an intraindividual comparison due to the common lack of premorbid test results. It can however be a useful tool in long term follow-up to validate and objectify a patient’s individual progress over time, and may be considered for this purpose.

While cognitive performance assessed via NCT did not seem to differ significantly between patients reporting high versus lower levels of CS, we did observe a statistically significant association between the patient reported severity of CS and the PHQ-9 score, FSS, the physical and psychological health as well as the environment section of the WHOQoL-BREF (see Tables 2, 3). Patients complaining of hiCS were more likely to reach higher scores when screened for symptoms of depression, higher levels of fatigue and lower levels of physical and psychological health. Beyond, assured psychiatric diagnoses were more frequent in patients reporting hiCS, with clinical depression being the most common. In a different study, Stallmach et al. compared PC patients (n = 355) to sepsis survivors (n = 272) and found no significant difference in the prevalence of cognitive dysfunction (23.5% vs 21.3%), however, fatigue (93.2% vs 67.8%) and signs of depression (81.3% vs 10.9%) appeared to be more prevalent in the PC group [10]. Similarly, Nalbandian et al. describe a higher prevalence of psychiatric diseases, including mood disorders such as depression, after infection with SARS-CoV-2 compared to influenza or other respiratory pathogens [3]. A meta-analysis by Han et al. even revealed a pooled prevalence of depression and anxiety symptoms of up to 23% in PC patients [34]. With this in mind, the association of subjectively high (four-point Likert scale), but objectively rather mild (NCT) levels of CS with higher PHQ-9 scores may indicate that our PC patients describe and experience neurocognitive disability as more severe due to cognitive distortions (i.e. catastrophizing, polarization) as laid out in the cognitive model of depression [35, 36]. Beyond, mood disorders in general are known to negatively affect cognitive functions [37, 38] and could contribute to CS in respective PC patients or at least modulate their perception of their own cognitive abilities. Thus, the relatively high prevalence of mood disorders and depression in PC patients as laid out in the mentioned literature and indicated by our data may contribute to the pathogenesis behind CS. It should be stated (restrictively) that the screening tools applied by us (PHQ-9, WHOQoL-BREF) do not allow a formal diagnosis of depression or other mood disorders. Most of our patients’ assured psychiatric diagnoses had been acquired before SARS-CoV-2 infection, which implies that chronic mood disorders and other psychiatric conditions may increase the susceptibility of such patients for SARS-CoV-2 associated CS and/or modulate their perception of CS severity. Based on our findings, we suggest that in the future, different assessments for cognitive impairment such as the THINC-integrated tool (THINC-it) [39] may be explored in PC patients to cover cognitive domains commonly affected by depression [38], and that PC patients reporting symptoms of depression are considered for further psychiatric evaluation.

Another important finding of this study is the high frequency of sleep disorders in patients complaining of hiCS. Insomnia is known to contribute to cognitive impairment [40] as well as a reduced quality of life [41], and is a frequent symptom found in PC patients, the prevalence of which ranges from 12 to 78.6% in the published literature [34, 42, 43]. There is a heterogeneity of findings with regard to insomnia and its impact on various cognitive domains, including attention, memory and executive function; however, of all cognitive domains, concentration is reported to be most frequently impaired by insomnia patients [40]. Fittingly, results of the d2-R section addressing concentration capacity of our PC patients (Table 3, d2R—CC) showed the most pronounced impairment of all our NCT regarding the entire patient cohort, followed by assessments of attention (RBANS—DS and RBANS—SNT). Thus, sleep disturbance in PC patients should be considered as a possible cause of CS and concentration issues in particular, and may offer a point of action for psychotherapeutic and/or pharmaceutical treatments.

In our patient cohort, radiological assessments of the brain, mostly MRI scans, could not detect any structural abnormalities that could potentially explain the reported CS, with two exceptions being one case of newly diagnosed MS and another with WML. In a systematic review assessing 35 published reports, Shan et al. found that increased white matter hyperintensities and decreased gray matter volume may be MRI signs associated with CS in PC patients [12]. However, the authors were hesitant to draw causal conclusions. Another systematic review by Okrzeja et al. found hypometabolism in several areas of the frontal, temporal and parietal lobes as correlates for numerous neurocognitive symptoms using 18-fluorodeoxyglucose positron emission tomography (^18^F-FDG-PET) [44]. In contrast, the findings by MRI included a very wide range of possible signs, and numerous alterations were not related to the respective symptoms. Overall, the authors constitute a lack of structural changes specific for the PC condition. Together with these data, our findings indicate that routinely performed MRI or CT scans in PC patients complaining of CS often fail to identify neurostructural correlates due to a lack of clinically defined structural or functional abnormalities. We strongly encourage future studies to further explore possible neuroradiological correlates. Brain imaging should still be considered as a diagnostic option in PC patients with CS, especially if a different underlying cause for the cognitive complaints is suspected, needs to be ruled out, or if other neurological symptoms need to be assessed.

In summary, NCT revealed mild impairment of mainly the working memory, attention and concentration in PC patients reporting CS. It seems reasonable to assume that concomitant or pre-existing mood disorders and other psychiatric conditions may contribute to the pathogenesis of PC associated CS or at least modulate the patients’ perception of their own neurocognitive capabilities. Further, insomnia appears to be an important risk factor for PC patients to develop CS and should be addressed therapeutically if possible. In our study, radiological assessments of the brain failed to identify probable neurostructural correlates for the reported neurocognitive complaints but in two cases, while other studies using advanced radiological sequences have. Beyond, brain imaging did reveal abnormal findings in a considerable fraction of our patients, even though they are not likely to account for the reported cognitive symptoms and most likely were not related to COVID-19. Further studies and meta-analyses are needed to identify radiological approaches fit to assess cognitive symptoms in PC patients, and to determine their clinical implications. Lastly, PC patients should be evaluated in an interdisciplinary approach. These findings support the routine usage of NCT in the work-up of PC patients and underscore the importance of radiological imaging. An interdisciplinary assessment of PC patients including neurologic and psychiatric work-up appears advisable.

## Supplementary Information

Below is the link to the electronic supplementary material.

Supplementary file1 (PDF 263 KB)

## Data Availability

All clinical data will be made available upon reasonable request.
