# Supplementary material for: Characterization of cognitive symptoms in post COVID-19 patients
Source: Eur Arch Psychiatry Clin Neurosci. 2024 May 13;274(8):1923–34. doi: 10.1007/s00406-024-01821-z (PMC11579195; doi:10.1007/s00406-024-01821-z)
Supplement: Supplementary file 1 — Supplementary file1 (PDF 263 KB) [file 406_2024_1821_MOESM1_ESM.pdf]

Supplementary Materials

Article Title:

Characterization of Cognitive Symptoms in Post COVID-19 Patients

Journal:

European Archives of Psychiatry and Clinical Neuroscience (EAPCN)

Authors:

Michael Ruzicka, Simone Sachenbacher, Fides Heimkes, Aline Olivia Uebleis, Susanne Karch, Fabienne Grosse Wentrup, Gerardo Jesus Ibarra Fonseca, Nora Wunderlich, Johannes Bogner, Julia Mayerle, Michael von Bergwelt-Baildon, Peter Falkai, Marion Subklewe, Thomas Ruzicka, Christopher Benesch, Elisabeth Valdinoci, Anna Pernpruner, Anabel Thomas, Bernhard Heindl, Hans Christian Stubbe*, Kristina Adorjan*

*Contributed equally

Corresponding author:

Michael Ruzicka

University Hospital / LMU Klinikum, Department of Medicine III, LMU Munich

Marchioninistrasse 15, 81377 Munich, Germany

Email: michael.ruzicka@med.uni-muenchen.de

Phone: +49 (0)89 4400-0

Fax: +49 (0)89 4400 74994

Supplementary Figure 1. Density distribution of acute SARS-CoV-2 infections across time and by CS severity

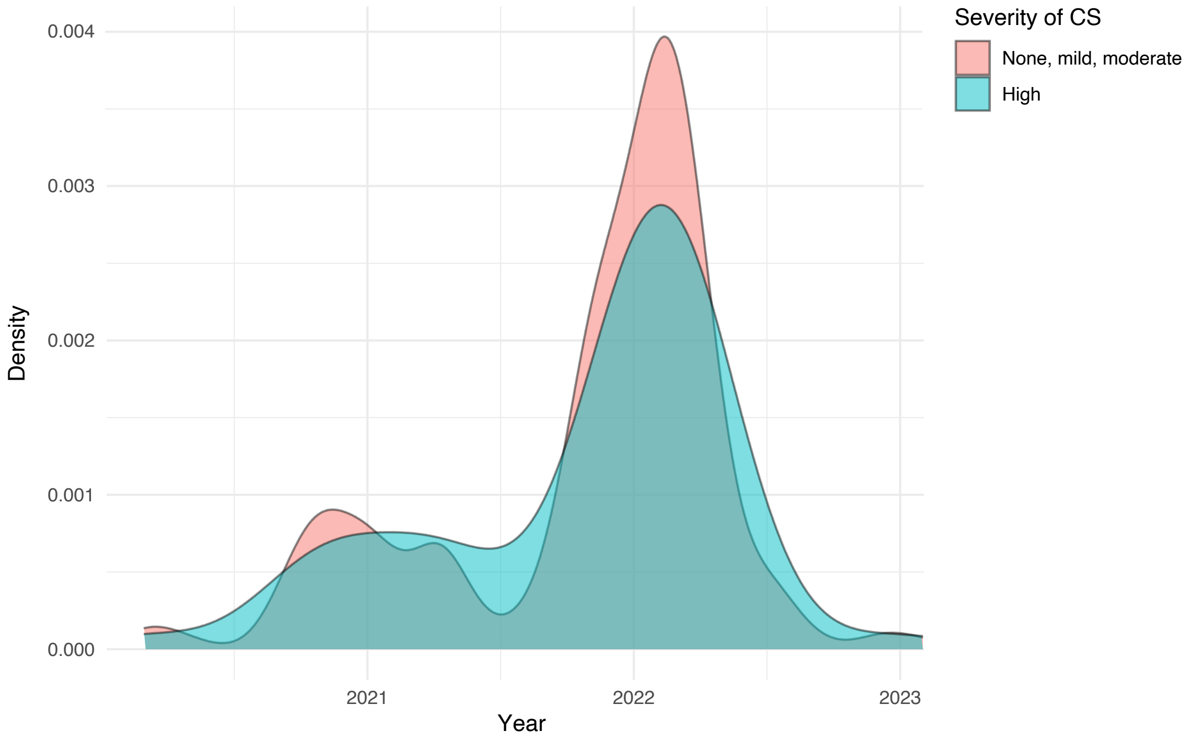

Supplementary Table 1. Neurocognitive assessments, respective abbreviations and cognitive domains assessed

Neurocognitive assessment	Abbreviation	Cognitive domain assessed
**Vocabulary test**	VT	Premorbid intelligence quotient (IQ)
**Trail Making Test – part A**	TMT-A	Visuomotoric speed
**Trail Making Test – part B**	TMT-B	Visuomotoric speed, cognitive flexibility & working memory
**Letter-Number-Span**	LNS	Working memory
**d2-R Test of Attention**	d2R	Concentration, attention
Concentration capacity	- CC	
Speed of operation	- Sp	
Accuracy	- Acc	
**The Repeatable Battery for the Assessment of Neuropsychological Status**	RBANS	
List Learning[1]	- LL	Immediate memory
Memorized Words in the 4th Round[2]	- MW4	
Story Memory[3]	- SM	
List Recall[4]	- LR	Delayed memory
List Recognition[5]	- LRg	
Story Recall[6]	- SR	
Figure Recall[7]	- FR	
Figure Copy[8]	- FC	Visuospatial function / construction
Line Orientation[9]	- LO	
Digit Span[10]	- DS	Attention
Coding / Symbol-Number-Test[11]	- SNT	
Semantic Fluency[12]	- SF	Language
Picture Naming[13]	- PN	

[1]Learning new words from a list of 10 words

[2]Memorized words in the fourth round

[3]Memorized details from a given story with 12 details

[4]Recalled words from a list of 10 after delay

[5]Number of words the patient can refer to from a previous list of words (n = 20) correctly

[6]Number of details the patient can remember from a story (with 12 details) after delay

[7]Memorizing a complex geometric image

[8]Patient is asked to draw a complex geometric image presented in front of them

[9]Patient is asked to recognize a certain angle out of multiple lines

[10]Patient is asked to repeat numerical sequences in the right order

[11]Patient is asked to name numbers related to symbols in a number-symbol-matrix

[12]Patient is asked to name as many names as possible of vegetables and fruit in 1 minute

[13]Patient is asked to name the items on ten pictures

Supplementary Table 2. Number of patients below -1SD of each NCT subtest

NCT subtest	**Number of patients below -1SD** (n_{total} = 78)
TMT-A	23 (29.5%)
TMT-B	20 (25.6%)
LNS	25 (32.9%)
d2-R - CC	34 (45.3%)
d2-R - Sp	8 (10.5%)
d2-R - Acc	14 (20.9%)
**RBANS - LL**	4 (5.1%)
**RBANS - MW4**	2 (2.6%)
**RBANS - SM**	12 (15.4%)
**RBANS - LR**	1 (1.3%)
**RBANS - LRg**	22 (28.2%)
**RBANS – SR**	12 (15.4%)
**RBANS - FR**	9 (11.5%)
**RBANS - FC**	27 (34.6%)
**RBANS - LO**	4 (5.1%)
**RBANS - DS**	37 (47.4%)
**RBANS - SNT**	34 (43.6%)
**RBANS - SF**	20 (25.6%)
**RBANS - PN**	12 (15.4%)

Supplementary Table 3. RBANS results of patients who reported hiCS vs mild to moderate CS

Reported intensity of cognitive symptoms	High (n = 26)	Mild or moderate (n = 52)	p value
**RBANS - LL**	110 [96.5;125]	113 [106;123]	0.478
**RBANS - MW4**	106 [95.0;108]	108 [104;108]	0.192
**RBANS - SM**	97.5 [89.2;112]	100 [89.8;107]	0.986
**RBANS - LR**	107 [101;112]	110 [102;122]	0.477
**RBANS - LRg**	85.0 [75.2;91.5]	89.0 [85.0;95.0]	0.192
**RBANS – SR**	93.0 [87.2;105]	100 [92.8;104]	0.598
**RBANS - FR**	103 [94.5;111]	104 [94.0;114]	0.598
**RBANS - FC**	87.0 [80.5;98.0]	91.0 [82.0;107]	0.478
**RBANS - LO**	104 [92.5;120]	108 [99.8;122]	0.590
**RBANS - DS**	80.5 [72.2;89.8]	92.0 [78.8;102]	0.192
**RBANS - SNT**	84.0 [68.2;98.5]	93.0 [82.0;108]	0.192
**RBANS - SF**	98.0 [88.2;103]	98.0 [81.8;114]	0.985
**RBANS - PN**	106 [81.0;107]	106 [106;106]	0.986

All results are depicted as age normalized standardized values (ASV) with a norm value of 100.
